# Current management strategies of urachal anomalies in pediatric patients: A scoping review

**DOI:** 10.3389/fruro.2023.1159439

**Published:** 2023-03-17

**Authors:** Yasmine S. Ghattas, David G. Gelikman, Kristen R. Ibanez, Pamela Ellsworth, Abhishek Seth

**Affiliations:** ^1^ College of Medicine, University of Central Florida, Orlando, FL, United States; ^2^ Division of Urology, Nemours Children's Health System/Nemours Children's Hospital, Orlando, FL, United States

**Keywords:** pediatrics - children, urachal abnormalities, management, literature review, urachus, infection

## Abstract

**Introduction:**

Management of urachal anomalies in pediatric patients has historically lacked a clear consensus between conservative and surgical management. We aimed to review and summarize the literature on the diagnosis, symptoms, and evolution in the management of urachal anomalies in pediatric patients.

**Methods:**

We performed a scoping literature review of PubMed/Medline and WebOfScience from January 2000 to February 2022.

**Results:**

32 publications were selected for inclusion in this analysis. 1,438 unique studies were identified with 32 studies meeting inclusion criteria. 15/32 studies discussed both conservative and surgical management, 14/32 studies discussed only surgical management outcomes, and 3/32 studies discussed diagnostic methods. The studies discussing conservative management supported the treatment of urachal anomalies with an initial conservative approach, which includes watchful waiting, repeated ultrasounds, lesion measurement, and antibiotic use. 5/32 of the included studies identified patients that were converted from conservative to surgical management with conversion rates ranging from 12.5% to 43.5% per study. 14/20 converted patients were identified to have a urachal cyst and 13/20 had a persistent infection.

**Conclusions:**

Strong evidence exists that supports initial conservative management over surgical management of pediatric urachal anomalies. However, predictive factors for determining which patients will require surgical management remain elusive. Treatment algorithms can potentially be developed once carefully developed prospective studies delineate statistically significant patient factors which necessitate surgical management over observation.

## Introduction

**Figure 1:**
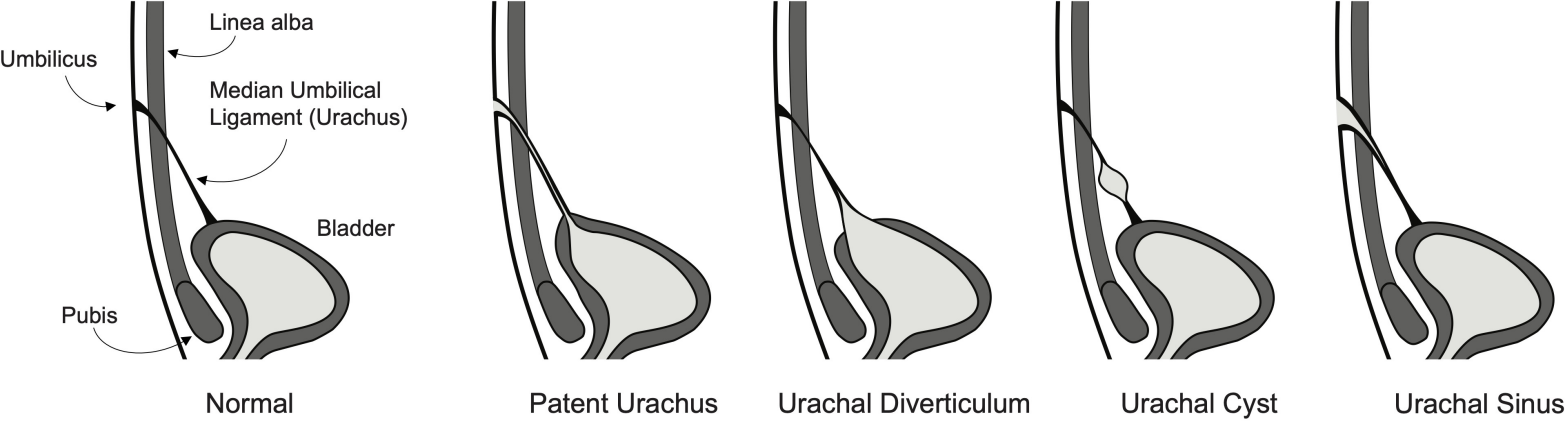
Illustration of a normal fully developed bladder with urachus and four of the most common urachal anomaly subtypes including patent urachus, vesicourachal diverticulum, urachal cyst, and umbilical-urachal sinus.

The urachus is a connection between the early fetal bladder and the allantois that aids in the removal of nitrogenous waste through the umbilical cord and placenta during gestation. This connection typically obliterates during fetal development or early in the neonatal course, forming the median umbilical ligament (1, 2). This lumen may fail to obliterate, thus leading to the abnormal persistence of an embryonic urachal remnant. Urachal remnants can be subtyped into different morphological anomalies including a patent urachus which has a fully patent lumen between the bladder and umbilicus, a vesicourachal diverticulum which contains a blind pouch attached to the bladder, a urachal cyst which contains a small patent lumen with two closed ends, and an umbilical-urachal sinus which contains a patent lumen at the umbilicus but does not extend to the bladder (**Figure 1**) (3). These four subtypes are classically reported in the literature, however, they may be further subdivided into anatomical variants based on length and connection with the lateral umbilical ligaments (4).

Urachal anomalies (UAs) were historically thought to be a rare occurrence, but modern increased utilization of imaging studies has revealed that UAs are more common than previously recorded. In 2015, Gleason et al. reported a 1.03% prevalence in the general pediatric population (5). Urachal malformations have varied clinical presentations, from asymptomatic (as the most common) to presenting with multiple non-specific symptoms including umbilical leakage, fever, abdominal pain, or acute/recurrent infection of the structure (6). Current management strategies for UAs include non-operative, conservative management in which the patient is followed to ensure symptoms do not worsen or recur, as well as surgical management in which the structure is removed, either in an open, laparoscopic, or robotically-assisted laparoscopic approach. The lack of a clear consensus on preferred management continues to persist due to the rarity of these defects and a paucity of existing data. Therefore, both approaches continue to be utilized. In this study, we aim to review the literature and identify current management strategies for pediatric patients with UAs.

## Materials and methods

### Search strategy

The following keywords were used to search the entire PubMed database on our search date of February 29, 2022, as follows: (urachal remnant) OR (urachal anomaly) OR (urachal abnormality) OR (patent urachus) OR (urachal diverticulum) OR (urachal sinus) OR (urachal cyst); these search terms were adjusted for use in Web of Science. Duplicates, articles published before 2000, and studies published in non-English languages were then removed using automated PubMed and WebOfScience filters.

### Inclusion and exclusion criteria

**Figure 2:**
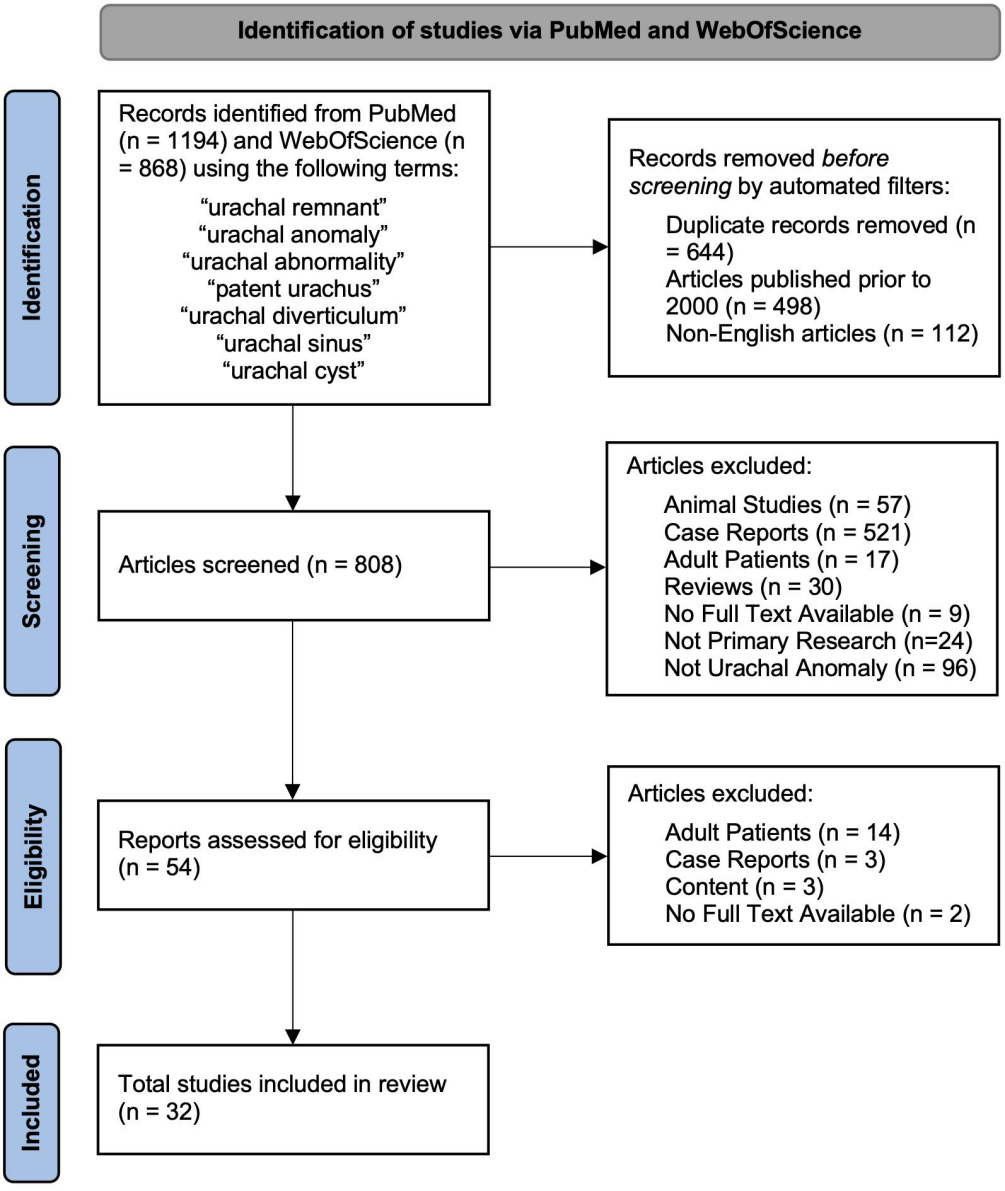
Flowchart of identification, screening, eligibility, and inclusion of studies in final analysis.

Published original research was analyzed to evaluate the diagnosis and management of UAs in pediatric patients. Studies were included if they contained a group of pediatric patients with a diagnosed UA and data available on patient age, diagnostic techniques, and management. To focus on more recent management strategies, only articles published during or after the year 2000 were included. The abstract and title of the remaining studies were screened by two medical student independent reviewers (YG and DG) and the decision to include and exclude studies was taken in consensus with pediatric urology faculty. **Figure 2** summarizes the article identification process. Studies were excluded if they fell into the following categories: animal studies, case reports, adult population included (>18 years of age), reviews, lack of abstract and full text, non-primary research (i.e., systematic review, literature reviews, etc.), and articles not primarily focused on UAs and their management. All studies that met the inclusion criteria were utilized to minimize study selection bias. Articles that remained were read in their entirety.

### Study identification and characteristics

Of the 1,418 unique studies identified through the initial literature search, 32 were ultimately included in this review as described in **Figure 2** along with reasons for exclusion. The characteristics of each individual study are listed in **Appendix 1** including the study type, number of patients, diagnostic imaging, type of UA present (if specified), and management strategy. These characteristics are summarized in **Table 1**.

**Table 1:** Characteristics of included studies and patient population.

Study Type	Number of Studies (N)	Percentage of Studies (%)	
Retrospective Chart Review	30^†^	93.75	
Retrospective Database Review	1	3.13	
Randomized Controlled Trial	1	3.13	
Prospective Surgical Study	1^†^	3.13^†^	
Total Number of Included Studies	32		
Patient Management	Number of Studies (N)	Percentage of Studies (%)	
Surgical Only	18	56.25	
Surgical & Conservative	14	43.75	
Conservative Only	0	0	
Diagnostic Imaging Used	Number of Studies (N)	Percentage of Studies (%)	
US	29	90.63	
CT	13	40.63	
VCUG	11	34.38	
MRI	5	15.63	
Other (i.e., Sinogram, Fistulography, Cystography, etc.)	5	15.63	
Patient Sex	Number of Patients (N)	Percentage of Patients (%)	
Male	805	37.08	
Unspecified	773	35.61	
Female	590	27.18	
Total Number of Patients	2,171		
Urachal Anomalies Present	Number of Studies (N)	Number of Patients (N)	Percentage of Patients (%)
Unspecified Anomaly	19	1657	76.32
Urachal Cyst	19	239	11.01
Patent Urachus	12	112	5.16
Urachal Sinus	12	100	4.61
Urachal Diverticulum	4	19	0.88
Urachal Fistula	2	18	0.83
Urachal Duct	2	16	0.74
No Anomaly	1	10	0.46
Total Number of Patients		2,171	
Types of Surgical Management	Number of Studies (N)	Number of Patients (N)	Percentage of Patients (%)
Open	13	643	57.67
Unspecified	10	250	22.42
Laparoscopic	14	189	16.95
Robotic	3	33	2.96
Total Number of Patients Treated Surgically		1,115	

^†^Ueno et al. (1) was classified as both a prospective surgical study and retrospective chart review.

US, ultrasound; CT, computed tomography; MRI, Magnetic Resonance Imaging; VCUG, voiding cystourethrogram.

## Results

Characteristics of the included studies, including study type, are noted in **Table 1** (1, 5, 7–36). One of the retrospective chart reviews also contained elements of a prospective surgical study where 8 patients were randomly assigned to receive surgical treatment (1). Three of the studies (9.4%) classified as retrospective chart reviews discussed diagnostic methods and reviewed imaging studies used in diagnosis without primarily focusing on the treatment of UAs (8, 25, 31).

Of the 32 included studies, 18 of them (56.3%) reviewed patients who were only treated surgically and discussed variations in surgical technique and management, while 14 of them (43.8%) reviewed patients who received either conservative, surgical, or combined management. None of the studies reviewed solely contained patients that were treated conservatively. Every study included at least one patient requiring surgical intervention. All the studies used at least one form of imaging to confirm the diagnosis of urachal remnants, with ultrasound (US) being used most (in 90.6% of studies), followed by CT (40.6%) and VCUG (34.4%).

There was a total of 2,171 patients across all 32 studies which includes 805 male patients (37.1%), 590 female patients (27.2%), and 773 patients (35.6%) of unspecified sex. Many studies did not list specific UA subtypes for patients, however, where specified, urachal cysts were the most common anomaly - found in 19 studies, comprising 239 total patients (11%). A patent urachus and urachal sinus were the next most common anomalies, found in 19 and 12 studies, comprising 112 (5.2%) and 100 (4.6%) patients, respectively. Every study had at least one patient with a surgically managed urachal remnant, making up a total of 1,115 patients who were treated surgically. Though many studies did not list the specific surgical approach, open surgery was the most common (in 643 patients) and made up 57.7% of all surgical cases. Unspecified surgical approaches made up 22.4% of cases, and laparoscopic and robotic-assisted laparoscopic approaches made up 17% and 3% of all surgical cases, respectively. Laparoscopic techniques mentioned in the literature began in 2007, and robotic techniques began in 2013.

When specified, the subtype of the UA was listed in descending order of prevalence for each study within **Appendix 1**. Most studies also included a distinction between symptomatic and asymptomatic (incidental finding) UAs, and this is listed in **Appendix 1** along with presenting symptoms in descending order of prevalence. Recommendations gathered from each included article are listed in **Appendix 1** and recommendations regarding imaging or surgical techniques are listed in **Appendix 1**

### Conversion from conservative to surgical management

Out of the 32 total studies, our review identified five studies in the past two decades that included patients who were initially tried on conservative therapy that failed, leading to surgical intervention. Within these studies, we identified a total of 20 patients that were converted to surgical management with conversion rates ranging from 13% to 44% per study (13, 19, 21, 24, 27). The conversion percentage, the reason for the conversion, specific UA involved, and surgical complications are listed for each study in **Table 2**. The conservative management tab lists the total number of patients who were initially tried on conservative management and the number of patients who were ultimately converted to surgical correction. In the surgical management tab, the total number of surgical patients listed includes all surgically managed patients in the study, including those who were converted from initial conservative management. Out of these patients converted to surgical treatment, 14 of them had a urachal cyst (70%), 1 patient had a urachal sinus (5%), and 5 patients had unspecified anomalies (25%). The most common reason for conversion was an infection, found in 13 patients (65%). Of the total 20 patients who converted from conservative to surgical management, all were recurrently symptomatic. 5 (25%) initially presented symptomatically and 15 (75%) were unspecified. 2 (10%) were males and 18 (90%) were of unspecified gender.

**Table 2:** Studies identifying conversion of patients from conservative to surgical management.

	Conservative Management				Surgical Management		
Reference	Total^a^ (N)	Converted* N (%)	Reason for Conversion = N (%)	Urachal Anomalies of Converted Patients = N (%)	Total^b^ (N)	Post-Surgical Complications	Study Total (N)
Dethlefs et al. (13)	24	3 (12.5%)	Infection = 1 (33%), Unspecified = 2 (67%)	Unspecified	47	Overall complications = 23 (48.9%)	68
Stopak et al. (19)	15	2 (13.3%)	Infection = 2 (100%)	Unspecified	72	Wound infections = 10 (13.9%) Persistent drainage = 2 (15.4%) Persistent granuloma = 1 (7.7%)	85
Lipskar et al. (24)	10	3 (30%)	Infection = 3 (100%)	Urachal cyst = 3 (100%)	8	Unspecified	15
Nogueras-Ocaña et al. (21)	12	2 (16.7%)	Infection = 2 (100%)	Urachal cyst = 2 (100%)	3	Unspecified	13
Galati et al. (27)	23	10 (43.5%)	Infection = 5 (50%), Unspecified = 4 (40%) Failure to Resolve = 1 (10%)	Urachal cyst = 9 (90%), Urachal sinus = 1 (10%)	10	Unspecified	23
Total	84	20 (23.8%)	Infection = 13 (65%), Unspecified = 6 (30%) Failure to resolve = 1 (5%)	Urachal cyst = 14 (70%), Unspecified = 5 (25%) Urachal sinus = 1 (5%)	140		204

*Patients initially treated conservatively and later converted to surgical management.

a Total number of patients initially treated conservatively.

b Total number of patients ultimately treated surgically (including patients that converted from initial conservative treatment).

## Discussion

Originally, obliteration of the urachus was believed to be a strictly prenatal phenomenon, and thus persistence of any part of the urachus was thought to be pathological. In some reports from the early 1970s, it was recommended to remove all urachal remnants when discovered, even those discovered incidentally (3). This sentiment continued through the early 2000s, from 2000-2006, the recommended management of urachal anomalies was surgical excision regardless of symptom presentation, citing the potential for later infection or evolution into cancer. Even in 2006, Choi et al. supported surgical resection regardless of symptomatic status citing a lack of spontaneous involution and the potential for infection or evolution into cancer (30). In the past few decades, the more frequent use of advanced imaging studies (such as US) has increased the number of UAs diagnosed incidentally and provided insight regarding the presentation of these abnormalities, with the majority of UAs lacking any noticeable symptoms and containing a minimal risk of malignancy (1). With increased imaging data, studies have shown that involution of the urachus may even occur postnatally (1, 2). In 1998, Ziegler et al. demonstrated that a group of random, asymptomatic newborns all had a urachal remnant on US which spontaneously involuted on a second US exam in 3-5 months (2). In 2003, Ueno et al. corroborated spontaneous involutions in children up to 1 year of age, and was the earliest study to recommend conservative management, recommending non-invasive management for both symptomatic and asymptomatic presentations (1). In 2008 and 2009, the literature showed opposing recommendations for management. Three studies were published, with each giving a different suggestion for management: Yapo et al. continued to support surgical management regardless of symptom presentation (notably, the basis for excision of asymptomatic patients was due to a stated 51% chance of malignancy), Galati et al. supported conservative management for all asymptomatic patients and all symptomatic patients under 6 months of age, and Copp et al. stated an inability to provide recommendations based on inconclusive evidence (26–28).

Starting in 2010, the literature appears to address the inconsistency in management recommendations and sought to investigate the best course of management. While these most recent studies consistently support the use of conservative management for asymptomatic presentation. From this time point, it is debated which patients require surgery based on age and symptom presentation. In 2010, Lipskar et al. offered the first proposed treatment algorithm for distinguishing between surgical and conservative management for symptomatic patients, and based the algorithm on symptom presentation, type of UA, and whether UA resolution was achieved after antibiotic treatment (24).

Further studies found that the associated pathological findings are typically benign, and the calculated number needed to treat to prevent the risk of adenocarcinoma formation is large, at 5,721 persons (5). Thus, removal of asymptomatic remnants during the first year of life is likely unnecessary. Most remnants may be observed conservatively, with infected remnants initially receiving antibiotic treatment. If a patient fails conservative management and/or presents with severe symptoms, then surgical management should be considered. Surgical excision by an open approach has typically been the gold standard, but over the last two decades, laparoscopic and robotic approaches have shown to be safe and effective alternatives to open surgery with increased cosmetic advantages (9, 10, 12, 14–17, 22, 35). The current landscape of UA management consistently juggles conservative and surgical therapies, with no clear predictive signs or guidance on when patients who fail conservative treatment should be switched to definitive surgical management.

### Effectiveness of conservative treatment

In 2008, Galati et al. performed a retrospective chart review and discovered 23 patients with UAs (including 12 urachal cysts, 9 urachal sinuses, and 2 patent urachus) who were all initially managed conservatively with silver nitrate and/or unspecified length of observation (27). Urachal sinuses were more common in boys (8 out of 9) and urachal cysts were more common in girls (9 out of 12), while patent urachi occurred equally in both sexes. About half of the patients required surgical intervention and they recommended surgery for recurrent symptoms or for failure of involution after 6 months of follow-up. The length of time from the initial diagnosis to involution was not noted. Of the 10 patients who converted to surgical management, 9 had urachal cysts and 1 had a urachal sinus. In 2010, Lipskar et al. performed a retrospective chart review of 15 initially symptomatic patients of unspecified gender (24). The group was composed of 10 urachal cysts and 5 patent urachal sinuses. 10 patients were initially managed conservatively with infected urachal cysts receiving percutaneous drainage and antibiotics. 3 out of the 10 patients had recurrent infections and were converted to surgical management.

In a retrospective case series performed by Nogueras-Ocana et al. in 2014, 13 patients with UAs were identified, 12 of whom were initially managed conservatively with US imaging every 6 months for the first two years and annually thereafter (21). Two patients (both male, initially presenting symptomatically, and with urachal cysts) were converted to surgical treatment due to reinfection after an initial course of antibiotics. In patients who achieved spontaneous resolution, the time between diagnosis and resolution ranged from 2 to 24 months. In 2015, Stopak et al. performed a retrospective review of 85 patients with UAs which was followed up by a 68-patient retrospective review by Dethlefs et al. in 2019 to monitor the evolution of treatment within the same health system (13, 19). In the initial study, it was recommended that UAs be treated conservatively in the first 6 to 12 months of life, and surgically thereafter. Only 15 patients were managed conservatively, with 2 of them requiring surgical management. In the follow-up study, it was noted that care was better shifted to nonoperative management. 24 patients were managed conservatively, with only 3 patients ultimately requiring surgery.

Though we were able to identify five articles that contained patients who were converted from conservative to surgical management (13, 19, 21, 24, 27), the lack of consistency between these studies makes it difficult to ascertain salient features of patient demographics or presentations that are predictive of conversion to surgical therapy. The included studies did not have enough patients convert from conservative to surgical management to determine if there are predictive variables for those necessitating surgery such as: certain urachal remnant subtypes, presenting symptoms, age, or gender. In addition, although there were a few patients with noted symptomatic patent urachi who studies claimed to be treated with conservative management (27), their degree of patency was unknown, and further investigation into this specific anomaly subtype is required to determine the proper management of these patients. Surgical correction may be needed for treatment if this anomaly is persistently symptomatic (e.g., drainage of urine directly from the umbilicus) does not close spontaneously within the first few months of life.

To our knowledge, there has been no comprehensive review to date on UAs. This review identifies recent shifts in preference towards avoiding surgical management for patients under one year of age, with evidence absent for whether this suggestion in management remains true regardless of UA type, presenting symptoms, gender, premature birth status, or specific age. We are currently unable to identify patterns of patients that would be more likely to require a conversion to surgical management. Given the lack of information, we are unable to algorithmically decipher which patients are ideal candidates for surgery and which patients should be observed. Also, due to the lack of randomized controlled trials and prospective studies in the literature, we were also unable to determine an ideal conservative management strategy with included follow-up timelines. Thus it would be necessary to perform a large-scale, multi-site prospective study analyzing demographic information, UA subtypes, and symptomatology regarding different protocol strategies to determine the most efficacious treatment strategy for this pathology. Though, this is not feasible due to the relative rarity of complications and potential for conversion to adenocarcinoma (1).

## Conclusions

The studies included in this review support initial conservative management over prophylactic surgical excision of pediatric UAs, even in symptomatic cases. In the past few decades, a conservative approach has been increasingly favored, especially for the treatment of symptomatic remnants in children 6 months to 1 year of age, where spontaneous resolution and involution were likely, even if initially infected. In older patients, conservative treatment with antibiotics in infected cases has been shown to effectively treat symptoms – thereby eliminating the need for surgical intervention except for rare cases presenting with recurrent infections. There remains a question regarding which associated patient demographics, UA subtypes, or presenting symptoms predict the need for eventual surgical intervention. Currently available studies lack large enough sample sizes to draw any apt correlations. Without sufficient data from prospective studies, the authors are unable to provide a worthwhile management algorithm.

## Author contributions

YG, DG, KI, PE, and AS made substantial contributions to conception and design. YG, DG and KI performed acquisition and interpretation of data. YG, DG, KI drafted the article. YG, DG, KI, PE and AS revised the paper critically for important intellectual content. YG, DG, KI, PE, and AS approved of the final version to be published. All authors contributed to the article and approved the submitted version.
