# Supplementary material for: Current management strategies of urachal anomalies in pediatric patients: A scoping review
Source: Front Urol. 2023 Mar 17;3:1159439. doi: 10.3389/fruro.2023.1159439 (PMC12327296; doi:10.3389/fruro.2023.1159439)
Supplement: Supplementary file 1 [file DataSheet_1.pdf]

APPENDICES

Appendix 1. List of studies included in the systematic review along with diagnostic imaging used, anomalies recorded, and management.

No.	Reference	Year	Type of Study	N	Diagnostic Imaging	Anomalies (N, %)	Management (Conservative, Surgical, Both)
1	Nissen et al.	2022	Retrospective Chart Review	52	US	Patent urachus = 23(44%), Urachal sinus = 21(41%), Urachal cyst = 8(15%)	Both
2	Olthof et al.	2021	Retrospective Chart Review	81	US	Unspecified	Both*
3	Osumah et al.	2021	Retrospective Chart Review	23	US, CT, VCUG	Urachal cyst = 13 (56.5%), Urachal sinus =4 (17.4%), Patent urachus = 3 (13%), Unspecified = 3 (13%)	Surgical
4	Aylward et al.	2020	Retrospective Database Review	476	NA	Unspecified	Surgical
5	Hashizume et al.	2020	Retrospective Chart Review	7	US, CT	Urachal sinus = 3(42.9%), Patent urachus = 2 (28.6%), Urachal cyst = 1 (14.3%)	Surgical
6	Orbatu et al.	2020	Retrospective Chart Review	15	US	Urachal cyst = 7 (46.7%), Unspecified = 6 (40%), Patent urachus = 2(13.3%)	Both
7	Basuguy et al.	2019	Retrospective Chart Review	27	US, CT	Urachal cyst = 27 (100%)	Both
8	Dethlefs et al.	2019	Retrospective Chart Review	68	US, VCUG	Unspecified	Both
9	Tanaka et al.	2019	Retrospective Chart Review	30	US, CT, MRI	Unspecified	Surgical
10	Ahmed et al.	2017	Retrospective Chart Review	16	US	Unspecified	Surgical
11	Bertozzi et al.	2017	Retrospective Chart Review	23	US	Unspecified	Surgical

12	Sukhotnik et al.	2016	Retrospective Chart Review	8	US, CT	Urachal cyst = 5 (62.5%), Patent urachus= 3 (37.5%)	Surgical
13	Gleason et al.	2015	Retrospective Chart Review	721	US, CT, MRI, VCUG	Urachal remnants = 640 (89%), Urachal cysts = 66 (9%), Patent urachus = 11 (1.5%), Urachal diverticulum = 4 (0.6%)	Both
14	Sato et al.	2015	Retrospective Chart Review	27	US, CT, MRI, VCUG	Urachal sinus = 16 (59%), Urachal duct = 6 (22%), Urachal cyst = 5 (18%)	Both
15	Stopak et al.	2015	Retrospective Chart Review	85	US, CT, VCUG, Sinogram	Unspecified	Surgical
16	Bertozzi et al.	2014	Retrospective Chart Review	12	US	Unspecified	Surgical
17	Nogueras-Ocaña et al.	2014	Retrospective Chart Review	13	US, CT, Cystography	Urachal cyst = 9 (69.2%), Urachal fistula = 3 (23.1%) Urachal sinus = 1 (7.7%)	Both
18	Masuko et al.	2013	Retrospective Chart Review	5	US, MRI	Unspecified	Surgical
19	Naiditch et al.	2013	Retrospective Chart Review	103	US, CT, MRI, VCUG	Urachal cyst = 38 (36.9%), Patent urachus = 21 (20.4%), Unspecified = 20 (19.4%), Urachal diverticulum = 13 (12.6%), Urachal sinus = 11 (10.7%)	Both
20	Lipskar et al.	2010	Retrospective Chart Review	15	US, CT	Urachal cyst = 10 (66.7%), Patent urachus = 5 (33.3%)	Both
21	Sun et al.	2010	Randomized Controlled Trial	18	NA	Unspecified	Surgical

22	Widni et al.	2010	Retrospective Chart Review	53	US	Patent urachus = 29 (66%), Urachal sinus = 10 (19%), No urachal anomaly = 10 (19%), Urachal cyst = 4 (7%)	Surgical*
23	Copp et al.	2009	Retrospective Chart Review	29	US, VCUG	Unspecified	Both
24	Galati et al.	2008	Retrospective Chart Review	23	Radiographic	Urachal cyst = 12 (52.2%), Urachal sinus = 9 (39.1%), Patent urachus = 2 (8.7%)	Both
25	Yapo et al.	2008	Retrospective Chart Review	25	US, VCUG	Unspecified = 13 (52%), Urachal duct = 10 (40%), Urachal cyst = 2 (8%)	Surgical
26	Turial et al.	2007	Retrospective Chart Review	27	US	Urachal fistula = 15 (55.6%), Urachal cyst = 8 (29.6%), Urachal sinus = 4 (14.8%)	Surgical
27	Choi et al.	2006	Retrospective Chart Review	21	US, CT	Urachal cyst = 10 (47.6%), Patent urachus = 6 (28.6%), Urachal sinus = 4 (19%), Urachal diverticulum = 1 (4.8%)	Surgical
28	Little et al.	2005	Retrospective Chart Review	56	US, VCUG	Unspecified	Surgical*
29	Huang et al.	2003	Retrospective Chart Review	20	US, VCUG, Fistulography	Urachal sinus = 13 (65%), Patent urachus = 4 (20%), Urachal cyst = 3 (15%)	Both
30	McCollum et al.	2003	Retrospective Chart Review	26	US, VCUG, Cystoscopy, Sinogram	Unspecified	Surgical
31	Ueno et al.	2003	Retrospective Chart Review/Prospective Surgical Study	56	US	Unspecified = 50 (89.3%), Urachal sinus = 4 (7.1%), Vesicourachal diverticulum = 1 (1.8%), Urachal cyst = 1 (1.8%)	Both

32	Pesce et al.	2000	Retrospective Chart Review	10	US, CT	Urachal cyst = 10 (100%)	Surgical

*Though the types of management were recorded in these studies, the primary focus was on diagnostic imaging and authors did not comment on preferred management strategies of urachal anomalies

Abbreviations: US = ultrasound, CT = computed tomography, VCUG = voiding cystourethrogram, MRI = Magnetic Resonance Imaging

Appendix 2. List of presenting urachal anomaly symptoms

No.	Reference	Year	Symptomatic (N, %)	Presenting Symptoms
1	Nissen et al.	2022	Symptomatic = 52 (100%)	Umbilical discharge = 44 (85%), Umbilical erythema = 31 (60%), Abdominal pain = 9 (17%), Abdominal mass = 2 (4%), Dysuria = 1 (3%)
2	Olthof et al.	2021	Symptomatic = 81 (100%)	Umbilical discharge = 81 (100%)
3	Osumah et al.	2021	Symptomatic = 15 (65.2%), Incidental = 7 (30.4%)	Abdominal pain = 7 (46.7%), Umbilical drainage = 8 (53.3%), Fever = 5 (33.3%), UTI = 2 (13.3%), Abdominal mass = 1 (6.7%)
4	Aylward et al.	2020	Unspecified	Unspecified
5	Hashizume et al.	2020	Symptomatic = 7(100%)	Infection = 7 (100%), Umbilical discharge = 7 (100%)
6	Orbatu et al.	2020	Symptomatic = 8 (53.3%), Incidental = 7 (46.7%)	Abdominal pain = 3 (37.5%), Umbilical discharge = 3 (37.5%), Abdominal mass = 1 (12.5%)
7	Basuguy et al.	2019	Symptomatic = 27 (100%)	Abdominal pain =12 (54%), Umbilical discharge = 6 (28%), Abdominal mass = 2 (9%), Fever = 2 (9%)
8	Dethlefs et al.	2019	Symptomatic = 60 (88.2%), Incidental = 8 (11.8%)	Umbilical discharge = 52 (76.5%), Umbilical erythema/granulation tissue = 23 (33.8%) Infected cyst = 9 (13.2%),
9	Tanaka et al.	2019	Symptomatic = 30 (100%)	Unspecified

10	Ahmed et al.	2017	Symptomatic = 13 (81.3%),<br>Incidental = 3 (18.8%)	Infection = 5 (38.5%),<br>Drainage = 3 (23.1%),<br>Infection/Drainage = 2 (15.4%),<br>Dysuria = 1 (7.7%),<br>Hematuria = 1 (7.7%),<br>Pain =1 (7.7%)
11	Bertozzi et al.	2017	Symptomatic = 17 (73.9%),<br>Incidental = 6 (26.1%)	Umbilical discharge = 8 (47.1%),<br>Omphalitis = 7 (41.1%),<br>Abdominal pain = 6 (35.3%),<br>Stranguria = 3 (17.6%),<br>UTI = 1 (5.9%)
12	Sukhotnik et al.	2016	Symptomatic = 8 (100%)	Umbilical discharge = 8 (100%)
13	Gleason et al.	2015	Incidental = 667 (92.5%),<br>Symptomatic = 54 (7.5%)	Unspecified
14	Sato et al.	2015	Symptomatic = 27 (100%)	<u>Under 1 Year (n = 11):</u><br>Umbilical granulation = 6 (54%),<br>Umbilical discharge = 3 (27.3%),<br>Hernia = 1 (9.1%),<br>Umbilical Cord Cyst = 1 (9.1%),<br><u>Over 1 Year (n =12):</u><br>Abdominal pain = 12 (75%),<br>Discharge = 12 (75%),<br>Granulation = 2 (12.5%)<br>Abdominal mass = 2 (12.5%)
15	Stopak et al.	2015	Symptomatic = 81 (95%),<br>Incidental = 4 (5%)	Umbilical drainage = 43 (51%),<br>Umbilical polyp/granulation = 20 (24%),<br>Infection = 16 (19%),<br>Fistula = 1 (1%),<br>Umbilical cord cyst = 1 (1%)
16	Bertozzi et al.	2014	Symptomatic = 13 (100%)	Abdominal pain = 6 (46.2%),<br>Omphalitis = 4 (30.8%),<br>Umbilical discharge = 4 (30.8%),<br>Stranguria = 3 (23.1%),<br>UTI = 1 (7.7%),<br>Pyuria = 1 (7.7%)
17	Nogueras-Ocaña et al.	2014	Symptomatic = 9 (69.2%),<br>Incidental = 4 (30.8%)	Umbilical discharge = 5 (55.6%),<br>Omphalitis = 4 (44.4%),<br>Abdominal pain = 3 (33.3%),<br>Fever = 3 (33.3%)
18	Masuko et al.	2013	Symptomatic = 4 (80%),<br>Incidental = 1 (20%)	Omphalitis =3 (75%),<br>Hematuria = 1(25%)

19	Naiditch et al.	2013	Symptomatic= 78(75.7%),<br>Incidental = 18 (17.5%),<br>Unspecified = 7(6.8%)	Umbilical drainage = 60 (58.3%),<br>Fever/erythema/possible infection = 5 (4.9%),<br>Pain = 4 (3.9%),<br>Hematuria = 1 (1%),<br>Urinary retention = 1 (1%),<br>UTI = 1 (1%)
20	Lipskar et al.	2010	Symptomatic = 15 (100%)	Umbilical drainage = 10 (66.7%),<br>Abdominal pain = 6 (40%),<br>Omphalitis = 4 (26.7%),<br>Fever = 4 (26.7%),<br>Abdominal mass = 3 (20%),<br>Dysuria = 1 (6.7%),<br>Recurrent UTI = 1 (6.7%)
21	Sun et al.	2010	Symptomatic = 18 (100%)	Recurrent infections = 18 (100%)
22	Widni et al.	2010	Symptomatic = 53 (100%)	Umbilical discharge = 41 (77%),<br>Umbilical granuloma = 23 (43%),<br>Inflamed umbilicus = 14 (26%),<br>Abdominal pain = 8 (15%),<br>UTI = 4 (7.5%)
23	Copp et al.	2009	Symptomatic = 24 (82.8%),<br>Incidental = 5 (17.2%)	Umbilical discharge = 20 (83.3%),<br>Omphalitis = 10 (41.7%),<br>UTI = 1 (4.2%)
24	Galati et al.	2008	Symptomatic = 21 (91.33%)<br>Incidental = 2 (8.7%)	Umbilical drainage = 15 (65.2%),<br>Abdominal pain = 1 (4.3%),<br>Irritative voiding = 1 (4.3%),<br>Urinary retention = 1 (4.3%),<br>Fever = 2 (8.8%)
25	Yapo et al.	2008	Symptomatic = 24 (96%),<br>Incidental = 1 (4%)	Polyp granuloma = 16 (64%),<br>Umbilical discharge = 4 (16%),<br>Abdominal pain = 2 (8%),<br>Umbilical cellulitis = 2 (8%),
26	Turial et al.	2007	Symptomatic = 27 (100%)	Unspecified
27	Choi et al.	2006	Symptomatic = 21 (100%)	Umbilical discharge = 10 (47.6%),<br>Abdominal mass = 9 (42.9%),<br>Abdominal pain = 3 (14.2%),<br>Dysuria = 1 (4.7%),<br>Fever = 1 (4.7%),<br>Frequency/nocturia = 1 (4.7%)
28	Little et al.	2005	Symptomatic = 56 (100%)	Umbilical drainage = 24 (43%),<br>Umbilical infection = 24 (43%),<br>Abdominal mass/pain = 8 (14%)

29	Huang et al.	2003	Symptomatic = 20 (100%)	Umbilical discharge = 16 (80%),<br>Granulation = 2 (10%),<br>Erythema = 3 (15%),<br>Abdominal pain = 1 (5%)
30	McCollum et al.	2003	Symptomatic = 25 (96.1%),<br>Incidental = 1 (3.8%)	Infection = 11 (42%),<br>Umbilical drainage = 7 (27%),<br>Pain = 3 (12%),<br>Umbilical polyp/granulation = 3 (12%),<br>Recurrent UTI = 1 (4%)
31	Ueno et al.	2003	Incidental = 36 (64.3%),<br>Symptomatic = 20 (35.7%)	Omphalitis = 17 (85%),<br>Hemo/proteinuria = 2 (10%),<br>Abdominal pain = 1 (5%)
32	Pesce et al.	2000	Symptomatic = 10 (100%)	Fever (100%),<br>Infraumbilical mass (100%),<br>Lower abdominal pain (50%),<br>Urinary complaints (40%),<br>Umbilical erythema (30%),<br>Umbilical discharge (10%)

Appendix 3. Management recommendations listed by each included article

	Management Recommendation		Overall Recommendation or Major Findings
**Study**	**Symptomatic ***	**Asymptomatic**	
2000 Pesce et al.	Surgical	Surgical	Complete excision repair regardless of presentation.
2003 Ueno et al.	Conservative	Conservative	No follow-up required for asymptomatic cases and no surgical management necessary unless multiple episodes occur, especially for patients under 1 year of age.
2003 McCollum et al.	Surgical	Surgical	Complete excision repair regardless of presentation.
2003 Huang et al.	Surgical	Surgical	Complete excision repair regardless of presentation.
2005 Little et al.	Surgical	No recommendation given	Surgical repair for symptomatic patients, no recommendation for asymptomatic patients due to lack of patients.
2006 Choi et al.	Surgical	No recommendation given	Surgical repair for symptomatic patients, no recommendation for asymptomatic patients due to lack of patients.
2008 Galati et al.	Conservative if < 6 months	Conservative	Excision if urachal remnant symptoms persist or fails to resolve after 6 months of age.

	of age, Surgical if > 6 months of age		
2008 Yapo et al.	Surgical	Surgical	Complete excision repair regardless of presentation.
2009 Copp et al.	No recommendation given	Inconclusive	Authors stated no recommendation for management of asymptomatic patients could be determined based on data obtained.
2010 Lipskar et al.	Varies by presentation	No recommendation given	Initial conservative treatment for infected urachal cysts and patent urachus, Surgical for patent urachus with cyst. Conservative management of infected cysts following drainage is a reasonable approach and cysts may obliterate over time.
2013 Naiditch et al.	Varies by presentation	Conservative	Non-specific atretic remnants, even when potentially symptomatic, may also be managed expectantly. Other types of URs may be managed expectantly on a case by case basis. ~15% postoperative complication must be taken into consideration when contemplating elective UR excision.
2013 Nogueras-Ocaña et al.	Conservative	Conservative	Initial conservative management of symptomatic UAs with antibiotics with transition to surgical management due to urachal cyst reinfection or persistent urachal sinus.
2015 Gleason et al.	Varies by presentation	Varies by presentation	Treatment of UAs should be considered on a case-by-case basis, with consideration for surgical operation taking into account evidence for low risk of malignancy.
2015 Stopak et al.	Varies by presentation	Conservative if < 1 year of age, no recommendation if > 1 year of age	Most UAs can be managed non-operatively for the first 6–12 months of life. Early intervention (< 6 months) should be reserved for patient with documented urine draining from the urachus or a documented abscess. Additionally, should patients require surgical excision, pre-operative antibiotics should be administered given the rate of post-operative infections.
2015 Sato et al.	Varies by presentation	No recommendation given	Conservative follow-up is recommended for UAs under 1-year old except if there are severe or repeated infections.
2017 Bertozzi et al.	Varies by presentation	Varies by presentation	Symptomatic cases should be considered on a case by case basis. The authors affirm that recent trends suggest conservative approach for asymptomatic

			cases, but decision should ultimately be made with the parents due to unknown risk of malignancy.
2019 Dethlefs et al.	Conservative	Conservative	No increase in adverse outcomes occurred due to a center-wide shift towards conservative management. Pre-operative antibiotics did not improve infection complication rates in surgical patients.
2019 Basuguy et al.	Surgical	Conservative	Patients with large cysts do not achieve any clinical or radiological resolution; therefore, those patients should undergo excision by laparotomy or laparoscopy.
2020 Orbatu et al.	Varies by presentation	Conservative	Conservative management is safe for asymptomatic patients. Surgical management should be reserved for patients with abdominal pain leading to acute abdomen, bladder rupture, patent urachus cases resistant to antibiotic treatment, and for large cysts.
2020 Aylward et al.	Conservative if < 1 year of age and minimally symptomatic	Conservative	Operative management of younger patients resulted in greater risk of reoperation, readmission, and longer length of stay. Given that nonoperative management is effective, it may be of benefit to delay resection of urachal remnants to after 1 year of age.
2022 Nissen et al.	No recommendation given	No recommendation given	Symptomatic UAs treated surgically showed an age-dependent complication pattern with more complications occurring patients over the age of 1.

*Management of initial presentation of symptoms

Appendix 4. Imaging and surgical technique recommendations from selected articles

	Study	Recommendation
Studies refining choice of imaging technique		
	2009 Widni et al.	US should be utilized as a first line diagnostic tool.
	2020 Othof et al.	The diagnostic accuracy of the abdominal ultrasound for detecting underlying causes for umbilical discharge needing surgical exploration in the pediatric population is relatively low even when performed by experienced pediatric radiologists.
Studies refining surgical management		
	2007 Turial et al.	Laparoscopic excision is a safe technique.
	2010 Sun et al.	Laparoscopic excision is a safe technique.
	2013 Makuso et al.	Laparoscopic excision is a safe technique.
	2014 Bertozzi et al.	Laparoscopic excision is a safe technique for symptomatic patients requiring removal that cannot be treated conservatively.
	2016 Sukhotnik et al.	Laparoscopic excision is a safe technique for symptomatic patients.

	2017 Ahmed et al.	Robot assisted laparoscopic excision is a safe and effective technique.
	2019 Tanaka et al.	OA and LA are safe and have similar effectiveness. Authors recommend choosing the surgical approach according to the surgeon's experience, patient's age, and hospital's specialty. LA is recommended for children aged ≥10 years due to cosmetic advantage.
	2020 Hashizume et al.	Complete laparoscopic removal of symptomatic urachal remnants and medial umbilical ligaments was a safe and effective minimally invasive approach, with better cosmetic outcomes.
	2020 Osumah et al.	Robotic assisted laparoscopic excision is a safe and effective technique and use of hidden incision endoscopic surgery techniques have short hospital stay length, low complication rates and low narcotic requirements.
